# Incidentally detected papillary thyroid carcinoma does not impair pregnancy outcomes during fertility treatment

**DOI:** 10.1210/jendso/bvag189

**Published:** 2026-08-13

**Authors:** Ayako Hoshiyama, Haruhiko Yamazaki, Jaeduk Yoshimura Noh, Natsuko Watanabe, Nami Suzuki, Miho Fukushita, Masako Matsumoto, Ai Yoshihara, Kenichi Matsuzu, Kiminori Sugino, Koichi Ito

**Affiliations:** Department of Internal Medicine, Ito Hospital, Tokyo 150-8308, Japan; Department of Surgery, Ito Hospital, Tokyo 150-8308, Japan; Department of Internal Medicine, Ito Hospital, Tokyo 150-8308, Japan; Department of Internal Medicine, Ito Hospital, Tokyo 150-8308, Japan; Department of Internal Medicine, Ito Hospital, Tokyo 150-8308, Japan; Department of Internal Medicine, Ito Hospital, Tokyo 150-8308, Japan; Department of Internal Medicine, Ito Hospital, Tokyo 150-8308, Japan; Department of Internal Medicine, Ito Hospital, Tokyo 150-8308, Japan; Department of Surgery, Ito Hospital, Tokyo 150-8308, Japan; Department of Surgery, Ito Hospital, Tokyo 150-8308, Japan; Department of Surgery, Ito Hospital, Tokyo 150-8308, Japan

**Keywords:** papillary thyroid carcinoma, surveillance, fertility treatment, pregnancy outcomes

## Abstract

**Background:**

Papillary thyroid carcinoma (PTC) is more common among women, including those of reproductive age. While a cancer diagnosis may influence pregnancy planning and treatment decisions in women receiving fertility treatment, the impact of PTC management on pregnancy outcomes remains unclear. We aimed to evaluate the clinical course of PTC diagnosed during fertility treatment and to clarify whether pregnancy outcomes differ according to the initial management strategy, surveillance or surgery.

**Methods:**

We retrospectively analyzed reproductive-age women diagnosed cytologically with PTC prior to pregnancy, who were referred to Ito Hospital for thyroid evaluation between January 2013 and December 2019, during fertility treatment. PTC cases were classified according to the Japanese Thyroid Tumor Treatment Guidelines 2018. Patients were followed for clinical course and pregnancy outcomes through December 2024.

**Results:**

Among 17 713 women undergoing fertility evaluation, 240 were diagnosed with PTC. Median age was 38 years, and most cases were classified as very low-risk (59.6%), followed by low-risk (27.1%) and intermediate risk (13.3%), with no high-risk cases. Among 96 surveillance patients followed for ≥12 months, disease progression occurred in 8 patients (8.3%), including 1 case during pregnancy. Patients who achieved live birth were significantly younger than those who did not (*P* < .001). Management strategy (surgery or surveillance) was not associated with the likelihood of live birth (*P* = .1868).

**Conclusion:**

Both surveillance and surgery can be safely integrated into fertility care for women with reproductive-age PTC. Tumor progression was rare and pregnancy outcomes were primarily influenced by maternal age rather than treatment strategy.

Thyroid cancer is more common among women than among men, and is the second most common cancer diagnosed around the time of pregnancy (1, 2). Differentiated thyroid cancer, specifically papillary thyroid carcinoma (PTC), is the most common type of thyroid cancer (3). Studies have shown that the incidence of PTC in women rises rapidly with increasing age, peaking in the forties and progressing more rapidly in younger patients than in older ones (4, 5). The predominant age at diagnosis of thyroid cancer thus includes the age at which many individuals wish to have children, making clarification of the clinical features of thyroid cancer in this population an important issue.

Subclinical hypothyroidism and thyroid peroxidase antibody (TPOAb)-positive status have shown associations with fertility and increased rates of pregnancy loss (6-8). The American Thyroid Association recommends treatment for patients with thyroid-stimulating hormone (TSH) > 2.5 mIU/L in women seeking to become pregnant by assisted reproductive technology (ART) (9). Consequently, thyroid function tests are increasingly being performed in obstetric and fertility clinics, leading to more referrals to endocrinology for detailed evaluations, during which PTC is sometimes incidentally detected. In addition, women undergoing fertility treatment often face a limited window for conception, making cancer-related treatment decisions particularly impactful on pregnancy planning. We aimed to evaluate the clinical course of PTC in reproductive-age women incidentally diagnosed before pregnancy during fertility treatment and to examine whether pregnancy outcomes differed according to the initial management strategy, surveillance or surgery.

## Materials and methods

### Study design and subjects

**Figure 1:**
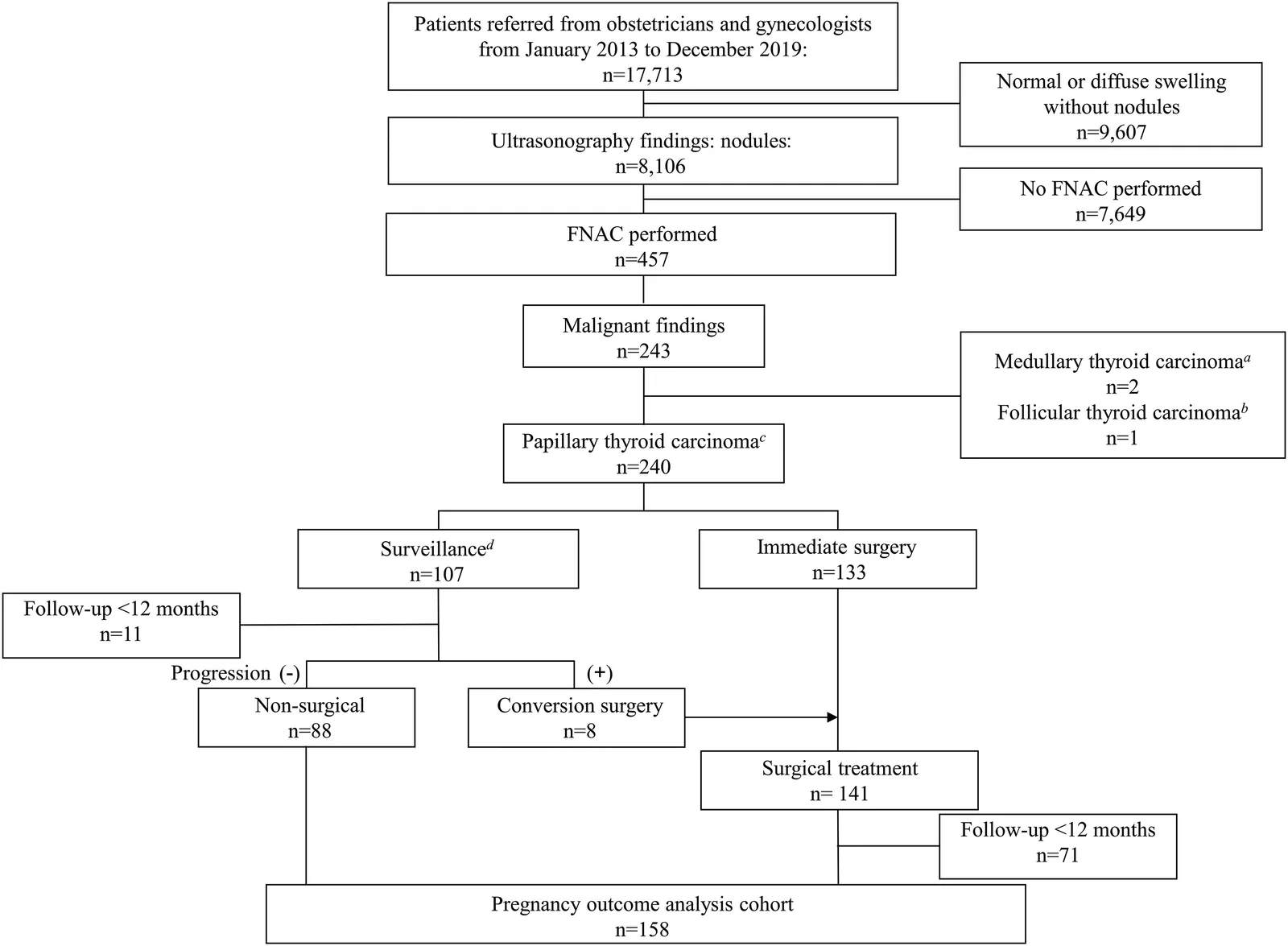
Patient selection and management. (A) All MTC cases showed cytopathological findings suggestive of MTC before surgery. (B) FTC was suspected from cytology and confirmed by postoperative histology. (C) PTC risk classification based on the guideline (12). (D) The Surveillance group primarily comprised 104 patients with very low-risk PTC, along with 3 patients showing low-risk tumors. FNAC, ultrasonography-guided fine-needle aspiration cytology; PTC, papillary thyroid carcinoma; MTC, medullary thyroid carcinoma; FTC, follicular thyroid carcinoma.

Participants in this study were adult women who had been referred to Ito Hospital (Tokyo, Japan), a center specializing in thyroid diseases, for the evaluation of thyroid function, based on the results of thyroid function tests conducted during gynecological examinations related to fertility treatment, between January 2013 and December 2019. Women undergoing fertility evaluation were identified based on referral information and other available clinical information. Medical records were reviewed in detail for all patients diagnosed with PTC. The patient selection process and study flow are shown in Fig. 1. All diagnostic evaluations and diagnoses were made before pregnancy. At the first visit, serum free triiodothyronine (FT3), free thyroxine (FT4), TSH, thyroglobulin antibody (TgAb), and TPOAb were measured as screening tests. Ultrasonography was performed to assess morphological abnormalities, including atrophy, hypoplasia, and other structural changes, to estimate the risk of future thyroid dysfunction and to guide appropriate monitoring strategies. Levothyroxine (LT4) supplementation was considered in patients with TSH levels above ∼2.5 mIU/L, including those with thyroid autoantibodies and those undergoing ART, with a target TSH level of <2.5 mIU/L, reflecting the practice adopted at our institution during the study period. The study data were collected before publication of the updated American Thyroid Association guidelines (10).

For patients showing thyroid nodules on thyroid ultrasonography, ultrasonography-guided fine-needle aspiration cytology (FNAC) was basically performed with reference to the criteria of the Japan Association of Breast and Thyroid Sonology (11). Only patients with a definitive cytological diagnosis of PTC were included and nodules with indeterminate cytology were excluded. Nodules diagnosed as PTC based on FNAC were classified into 4 risk categories according to the 2018 guidelines issued by the Japanese Society of Thyroid Surgery and the Japan Association of Endocrine Surgeons (12). Tumor staging was assessed based on the 8th edition of the tumor, node, metastasis classification of the International Union Against Cancer/American Joint Committee on Cancer (13).

Very low-risk PTC comprises tumors measuring ≤1 cm in diameter, without lymph nodes or distant metastases on imaging studies (T1aN0M0). Low-risk PTC is 1.1 to 2 cm in diameter, without lymph node or distant metastases (T1bN0M0). Intermediate-risk PTC indicates tumors not belonging to any of the very low-, low-, or high-risk classes. High-risk PTC includes tumors with at least one of the following clinical features: (1) tumor diameter >4 cm; (2) extrathyroidal extension to adjacent structures other than the sternothyroid muscle, or extranodal extension to adjacent structures from metastatic lymph nodes; (3) clinical node metastasis >3 cm; or (4) distant metastasis detected on imaging (12). Although assessments for metastases are typically conducted using computed tomography, imaging was omitted in this study due to concerns about undetected or potential pregnancies. Risk classification was therefore based solely on tumor and nodal status. Management strategies were primarily determined based on risk stratification, with consideration of patient preference. Patients were initially classified according to the initial management strategy into the immediate surgery group or the Surveillance group. For certain analyses, patients were also categorized according to the treatment actually received during follow-up. Those who underwent surgery at any time were included in the surgical treatment group, whereas those who did not undergo surgery during the study period were included in the nonsurgical group. The clinical course of patients diagnosed with malignant tumors was investigated from the time of diagnosis to the end of December 2024. Patients with follow-up duration <12 months were excluded from the analysis of disease progression, recurrence, and pregnancy outcomes. Information on pregnancy and childbirth was collected from patients through a structured questionnaire.

This study was approved by the ethics committee of Ito Hospital (approval no. 312). All methods were conducted in accordance with the *Ethical Guidelines for Medical and Health Research Involving Human Subjects in Japan* and the Declaration of Helsinki. An opt-out method was employed to allow patients the opportunity to decline participation.

### Assay

Thyroid function and antithyroid antibody testing were performed using an ECLusys electrochemiluminescence immunoassay kit (Roche Diagnostics, Basel, Switzerland). Reference ranges in our hospital were as follows: FT3, 2.2 to 4.3 pg/mL; FT4, 0.8 to 1.6 ng/dL; TSH, 0.2 to 4.5 mIU/L; TgAb, ≤40 IU/mL, and TPOAb, ≤28 IU/mL. TgAb and TPOAb were considered positive for values above the reference range.

### Statistical analysis

Statistical analyses were performed using JMP Pro 17 software (SAS Institute Inc., Cary, NC, USA). Data are presented as median and interquartile range (IQR) for continuous, nonparametrically distributed variables. LT4 dosages were rounded to the nearest 12.5-µg increment prior to analysis, to reflect actual prescription units. The Wilcoxon rank-sum test was used to compare continuous variables between 2 groups, and the χ^2^ test was used to evaluate categorical variables. Values of *P* < .05 were considered to indicate a statistically significant difference between groups.

## Results

### Baseline characteristics of patients with PTC

Among 17 713 women referred for thyroid evaluation, 240 (1.35%) were diagnosed with PTC based on cytological findings. The patient selection process is shown in Fig. 1. The baseline characteristics of these patients are summarized in Table 1, and characteristics of the overall study population are provided in Table S1 (14). Among patients with PTC, median age was 38 years (IQR, 35-41 years). The majority of cases were classified as very low-risk (59.6%), followed by low-risk (27.1%) and intermediate risk (13.3%), with no high-risk cases. Baseline thyroid function and autoantibody status are also shown in Table 1. Median duration of follow-up was 83 months (IQR, 69-102 months), with a minimum of 12 months and a maximum of 11 years.

**Table 1:** Baseline characteristics of patients with papillary thyroid carcinoma

Variable	
Patients, n	240
Age (years)	38 (35-41)
Tumor size (mm)	7.6 (5.2-12.9)
Risk category, n (%)	
Very low	143 (59.6)
Low	65 (27.1)
Intermediate	32 (13.3)
FT3 (pg/mL)	2.9 (2.7-3.1)
FT4 (ng/dL)	1.20 (1.10-1.30)
TSH (mIU/L)	2.70 (1.76-3.61)
TgAb-positive, n (%)	88 (36.7)
TPOAb-positive, n (%)	43 (17.9)

Values are given as median and IQR or number and percentage.

FT3, free triiodothyronine; FT4, free thyroxine; TSH, thyroid-stimulating hormone; TgAb, thyroglobulin antibody; TPOAb, thyroid peroxidase antibody.

### Initial management and clinical course of PTC

Patients were classified by initial management, with 105 very low-risk and 2 low-risk patients comprising the Surveillance group. The 143 patients with very low-risk PTC either continued observation (n = 105) or received surgical treatment (n = 38). Patients with very low-risk PTC underwent surgery for the following reasons: because the tumor was close to the recurrent laryngeal nerve in 9 patients; because the tumor was located adjacent to the trachea in 5 patients; and other reasons, physician judgment or patient preference in 24 patients. Family history of thyroid cancer was not systematically collected across multiple generations. However, a review of the medical records did not identify family history as a factor influencing treatment decisions. Table 2 summarizes the baseline clinical characteristics with PTC according to the initial management strategy in the immediate surgery group (n = 133) and surveillance group (n = 107). Median age was similar in the 2 groups, at 38 years. Median tumor size was generally larger in the immediate surgery group (12.2 mm, IQR 7.7-15.5 mm) than in the surveillance group (5.8 mm, IQR 3.9-7.0 mm), consistent with clinical selection criteria. Two patients in the surveillance group showed T1b, but surgery was postponed to prioritize the pregnancy. All other patients in the surveillance group had T1aN0M0 and were followed-up. Of the 96 PTC patients followed for more than 12 months, 8 (8.3%) initially underwent surveillance but subsequently required surgery due to disease progression. This was attributed to tumor growth in 5 patients and new lymph node metastasis in 3 patients, occurring 1 to 4 years after diagnosis (Fig. 1).

**Table 2:** Characteristics of the immediate surgery and surveillance groups

Variable	Immediate surgery (n = 133)	Surveillance (n = 107)
Age (years)	38 (35-41)	38 (35-41)
Tumor size (mm)	12.2 (7.7-15.5)	5.8 (3.9-7.0)
Risk classification, n (%)		
Very low	38 (28.6)	105 (98.1)
Low	63 (47.4)	2 (1.9)
Intermediate	32 (24.0)	0 (0.0)
High	0 (0.0)	0 (0.0)

Values are given as median and IQR or number and percentage.

Among the 133 patients in the immediate surgery group, 61 underwent surgery at our institution. Surgical procedures included lobectomy in 40 patients (65.6%) and total thyroidectomy in 21 (34.4%). Radioactive iodine (RAI) therapy was administered postoperatively to 5 of the 61 patients (8.2%). LT4 supplementation was required in 54 patients (88.5%), at a median dose of 75 μg/day (IQR 62.5-112.5 μg/day) (Table 3).

**Table 3:** Surgical and postoperative characteristics of patients who underwent surgery at our institution (n = 61)

Variable	Value
Type of surgery, n (%)	
Lobectomy	40 (65.6)
Total thyroidectomy	21 (34.4)
RAI therapy received, n (%)	5 (8.2)
Timing of pregnancy, n	
Before surgery	13
After surgery	18
LT4 supplementation, n (%)	54 (88.5)
LT4 dose, μg/day*^a^*	75 (62.5-112.5)

Values are given as number and percentage or median and IQR.

LT4, levothyroxine; RAI, radioactive iodine.

^*a*^Dose indicates the prescribed amount at the time of pregnancy for women who conceived, and the maximum dose during follow-up for those who did not.

### Pregnancy outcomes among PTC patients

During follow-up, 83 patients were not evaluable, mainly due to transfer to other hospitals. These included 11 patients in the Surveillance group and 71 in the surgical group, leaving 158 patients for analysis. Of these, 86 gave birth.

Median duration of follow-up was 81 months (IQR 66-103 months) in the surgical group and 85 months (IQR 71-102 months) in the nonsurgical group. Birth rates were 47.3% (26/55) in the surgical group and 58.3% (60/103) in the nonsurgical group, showing no significant difference (*P* = .2405). In the surgical group, among the 40 women who gave birth, 14 became pregnant before surgery and 26 became pregnant after surgery. LT4 supplementation was provided to 92.7% (51/55) in the surgical group, with a median dose of 75 μg/day (IQR 62.5-112.5 μg/day), while 79.6% (82/103) in the nonsurgical group received LT4, with a median dose of 50 μg/day (IQR 37.5-62.5 μg/day) (Table 4).

**Table 4:** Reproductive status and thyroid hormone supplementation in the surgical and nonsurgical groups

Variable	Surgical*^a^* (n = 55)	Nonsurgical (n = 103)	*P*-value
Age, years	39 (35-41)	38 (35-41)	.7375
Duration of follow-up, months	81 (66-103)	85 (71-102)	.6720
Patients with live birth, n (%)	26 (47.3)	60 (58.3)	.2405
LT4 supplementation, n (%)	51 (92.7)	82 (79.6)	.0389
LT4 dose (μg/day)	75 (62.5-112.5)	50 (37.5-62.5)	<.0001

Values are given as median and IQR or number and percentage.

LT4, levothyroxine.

^*a*^The surgical group included only women who conceived after surgery; those who conceived before surgery were classified into the nonsurgical group.

As shown in Table 5, patients who achieved live birth were significantly younger than those who did not (37 vs 40 years, *P* < .001). This age-related difference was observed regardless of treatment strategy. Consistent with this, choice of management (surgery or surveillance) was not associated with the likelihood of live birth (*P* = .1868). Other clinical factors, including LT4 supplementation status, LT4 dose, thyroid autoantibody positivity (TgAb or TPOAb), and TSH levels at first visit, did not differ significantly between live birth and nonlive birth groups. In addition, only 1 case of tumor enlargement during pregnancy and 1 case of postoperative recurrence were encountered, both of which were appropriately managed.

**Table 5:** Comparison of characteristics between patients with and without live birth

Variable	Live birth (n = 86)	Not live birth (n = 72)	*P*-value
Treatment			.1868
Surgical, n	26	29	
Nonsurgical, n	60	43	
Age (years)	37 (34-39)	40 (37-42)	<.001
LT4 supplementation			
n (%)	70 (81.4)	63 (87.5)	.2950
Dose (μg/day)	62.5 (37.5-75)	62.5 (50-75)	.5669
TgAb-positive, n (%)	49 (57.0)	39 (54.2)	.7232
TPOAb-positive, n (%)	26 (30.2)	17 (23.6)	.3517
TSH at first visit (mIU/L)	2.44 (1.59-3.71)	2.78 (1.98-3.72)	.3274

Values are given as median and IQR or number and percentage.

LT4, levothyroxine; TgAb, thyroglobulin antibody; TPOAb, thyroid peroxidase antibody; TSH, thyroid-stimulating hormone.

## Discussion

This single-center retrospective study conducted at a thyroid specialty hospital evaluated clinical features and pregnancy outcomes among women of reproductive age diagnosed with PTC during fertility treatment. Our results demonstrated that the presence of PTC and its treatment modality (as surgery or surveillance) did not significantly affect the likelihood of live birth. In contrast, younger maternal age was strongly associated with successful pregnancy outcomes, highlighting age as a key determinant of fertility in this population.

Among the 17 713 women undergoing fertility evaluation, PTC was identified in 1.35% of patients. This finding provides clinical context for PTC encountered during fertility treatment, where management decisions must balance oncologic and reproductive considerations. In this clinical setting, the characteristics and management of detected tumors are particularly relevant to reproductive planning. In the present cohort, most detected tumors were low or very low-risk, many of which were managed with surveillance. Only 8.3% of patients under surveillance experienced disease progression, and just 1 case (1.7%) progressed during or after pregnancy, with no distant metastases or cancer-related deaths. These findings support the safety of surveillance even during fertility treatment and pregnancy, in line with previous studies showing that pregnancy itself does not significantly accelerate PTC progression (5, 15).

Importantly, prior reports have shown that surgery can be safely delayed until after delivery, with even second-trimester surgery carrying little maternal or fetal risk (16, 17). Considering the low-risk of progression and the availability of safe treatment options after pregnancy, prioritizing surveillance and allowing pregnancy to proceed may be a reasonable and safe approach for women with low-risk PTC.

Surgery was performed in approximately half of the patients diagnosed with PTC, with most undergoing lobectomy. Postoperative RAI therapy was less commonly performed. The postoperative recurrence rate was low, and most patients were able to resume fertility treatment shortly after surgery. Although LT4 supplementation was more common and required higher doses in the surgical group, the difference did not appear to impact fertility outcomes. These data are reassuring and indicate that appropriate surgical management of PTC can be integrated into fertility care without significantly compromising reproductive goals.

In the present study, a proportion of T1a patients eligible for surveillance elected to undergo surgery, with patient preferences contributing to many of these decisions. Previous studies have shown that patients under active surveillance (AS), particularly women, may initially experience anxiety, although this tends to decrease over time (18, 19). In addition, quality of life has been reported to decline more in patients who have undergone surgery than in those managed conservatively (20). However, those studies did not specifically target women undergoing fertility treatment, a population that has distinct concerns, including potential treatment delays, concerns about pregnancy, and restrictions on fertility medication use. Some patients may choose surgery based on the anticipated duration of fertility treatment or whether in vitro fertilization is required.

Although AS has been considered safe even during pregnancy (16, 17), the implications in the setting of fertility treatment remain unclear. If surgery becomes necessary during prolonged fertility care, delaying pregnancy may lower birth rates due to advancing maternal age. Treatment decisions must often be made within a short timeframe before initiating fertility treatment, a period of heightened psychological stress. Further, some patients were referred elsewhere for earlier surgery, likely reflecting anxiety and a strong desire to prioritize cancer treatment. Nevertheless, pregnancy outcomes did not differ significantly between the surveillance and surgical groups, and tumor progression remained rare. These results indicate that surveillance can be safely considered even in women actively pursuing fertility, provided that individualized counseling is offered. Shared decision-making is essential to account for psychological burden, reproductive urgency, and patient preferences.

Currently, pregnancy-specific counseling for surveillance is insufficient. After appropriate explanation, patients should be offered the options of surgery and surveillance based on preference. The finding that PTC requiring surgery, including T1b tumors, does not adversely affect pregnancy, and that progression during surveillance is rare, provides important guidance for treatment planning. Further prospective studies are warranted to assess how AS influences fertility outcomes and psychological well-being.

Patients who achieved live births were clearly younger than those who did not, underscoring age as the primary factor affecting reproductive success (21, 22). This pattern was consistent across treatment strategies, with neither surgery nor surveillance affecting the likelihood of live birth. Other clinical factors, including LT4 supplementation and dose, thyroid autoantibody status, and TSH at first visit, likewise showed no significant differences and appeared to have minimal impact on pregnancy outcomes. Although TSH levels during pregnancy were not evaluated in this study, LT4 supplementation was adjusted based on TSH values in routine clinical practice and appeared to have minimal impact on pregnancy outcomes in this cohort.

Several limitations of the present study should be acknowledged. This was a single-center study at a thyroid referral hospital, which may limit generalizability. Detailed fertility treatment data were unavailable and some pregnancy outcomes were based on self-reported questionnaires, introducing potential reporting bias, particularly regarding early pregnancy loss. In addition, preoperative risk stratification relied primarily on ultrasonographic findings rather than final surgical pathology. No high-risk patients were included.

## Conclusion

Both surgery and surveillance can be safely integrated into fertility care for women with low-risk PTC. Pregnancy outcomes were not adversely affected by either approach or maternal age was a key determinant of live birth. These findings support individualized decision-making based on clinical context and patient values.

## Data Availability

The datasets analyzed during this study are not publicly available due to patient privacy and institutional restrictions but are available from the corresponding author on reasonable request.
